# Effects of the radio electric asymmetric conveyer (REAC) on motor disorders: An integrative review

**DOI:** 10.3389/fmedt.2023.1122245

**Published:** 2023-02-27

**Authors:** Vinícius Gomes Machado, Ana Beatriz Sorgenfrei Brun, Elisangela Ferretti Manffra

**Affiliations:** ^1^ Health Technology Graduate Program, Pontifícia Universidade Católica do Paraná, Curitiba, Brazil; ^2^ Physiotherapy Under-Graduate Program, Pontifícia Universidade Católica do Paraná, Curitiba, Brazil

**Keywords:** rehabilitation, motor disorders, radio electric fields, electrophysiology, neurophysiology, endogenous bioelectric fields

## Abstract

**Introduction:**

The radio electric asymmetric conveyer (REAC) is a technology that has the purpose of restoring the cellular polarity triggering the rebalancing of the endogenous bioelectric field, which considering the neurological dysfunctions, affects the neural communication mechanisms. The studies published so far show that the REAC neuromodulation technology has positive effects in treating these dysfunctions, with the principles of endogenous bioelectricity as a basis to achieve these effects.

**Objectives:**

This study aims to review the literature that explored the effects of REAC protocols on motor control and to identify which mechanisms would be involved.

**Materials and methods:**

This integrative review considered studies that used REAC as a therapeutic intervention directed at human motor control and experimental research with animals that applied REAC to obtain effects related to motor behavior.

**Results:**

Ten articles were included, eight clinical and two experimental studies. The clinical studies used the neuro postural optimization (NPO) protocol in 473 patients, of which 53 were healthy subjects, 91 were Alzheimer's disease patients, 128 were patients with atypical swallowing, 12 subjects with neurological diseases, and 189 were without the specification of disease. The experimental studies used the antalgic neuromodulation and neurodegeneration protocols in animal models.

**Conclusion:**

The information integrated in this review made it possible to consider REAC technology a promising resource for treating motor control dysfunctions. It is possible to infer that the technology promotes functional optimization of neuronal circuits that may be related to more efficient strategies to perform motor tasks.

## Introduction

The radio electric asymmetric conveyer (REAC) technology aims to elicit cellular reprogramming activity and modulate adaptive responses in the body by restoring the balance of cellular endogenous bioelectric fields (1). It has been the target of studies in diverse areas of health sciences such as neurology (2, 3), orthopedics (4, 5), gerontology (6), cardiology (7), rheumatology (8) and histopathology (9–11). The basis for achieving the desired effects of REAC technology is the endogenous bioelectricity which affects neural and cellular processing mechanisms and, under pathological conditions, would be unbalanced (11). According to Rinaldi et al. (2011), the REAC technology, especially its neuromodulation protocols, allows the regulation and modulation of endogenous bioelectric fields and, in this way, promotes better functioning of neuronal circuits (12–14). In addition, a balanced electric field is fundamental for cellular metabolism since cell activity is directly linked to electricity in the intra and extracellular environments (5).

The REAC technology works through the interaction of the radioelectric field produced by the REAC device with the endogenous bioelectric fields produced by the cells. After establishing this interaction, a series of cellular ionic fluxes are generated, triggering transcriptional and signaling events that drive reprogramming and differentiation decisions of cells affected by this interaction (11, 15, 16).

Scientific research published to date has shown that REAC neuromodulation technology has positive effects on the treatment of central neurological disorders such as depressive syndromes (17, 18), anxiety (17–23), stress (2, 18, 19, 21–28), Parkinson's (9, 10) and Alzheimer's diseases (3, 29–32).

In addition, the effects of REAC protocols seem to be beneficial for slowing down the processes of neurodegenerative diseases (32) and the aging process (6, 33–36), which inextricably cause changes in motor control mechanisms.

Although some studies (3, 29, 31, 37–39) have investigated the impact of REAC protocols on motor control, the topic needs to be explored. It is known that neurological conditions that impair motor control possibly modify the electric field produced by neuronal circuits, altering the communication and integration of information (40). Motor control depends on the integration of somatosensory information in the supraspinal centers, and the REAC protocols might contribute to this process through the functional optimization of neuronal circuits (14, 38). Given this scenario, this study aims to review the literature that explored the effects of REAC protocols on motor control and to identify which mechanisms would be involved.

## Materials and methods

The present study is an integrative review (41). We have searched for scientific articles published in PubMed/MEDLINE, Bireme/Brisa, SCIELO, Capes Periodicals, Clinical Trials, Scopus, and IEEE databases without time restriction. The keywords used for the search were “radio electric asymmetric conveyer” and “REAC” without linguistic restrictions.

To be included in this review, the studies should have applied REAC protocols and observed outcomes related to motor control. Studies that did not provide information on the REAC protocol or describe how it was applied would be excluded.

The screening process began with the title analysis to verify the use of REAC protocols. After the initial screening, the selected studies were retained for analytical reading of the abstract and verification of eligibility criteria. Those potentially eligible were analyzed in full detail, and those that did not fit the eligibility criteria were excluded.

The information extracted from the included studies were: title, author, year, country of origin, and type of study. Descriptive data from clinical studies were: sample (number, age, and gender), assessed motor task, outcomes, and assessment methods. In experimental studies, the animal model, the model's characteristics, the motor task, and the outcome evaluation methods were considered. Additionally, information about REAC protocols was extracted from both clinical and experimental studies.

Two reviewers carried out the entire methodological process of this review and the inconsistencies would be resolved by a third reviewer.

## Results

**Figure 1:**
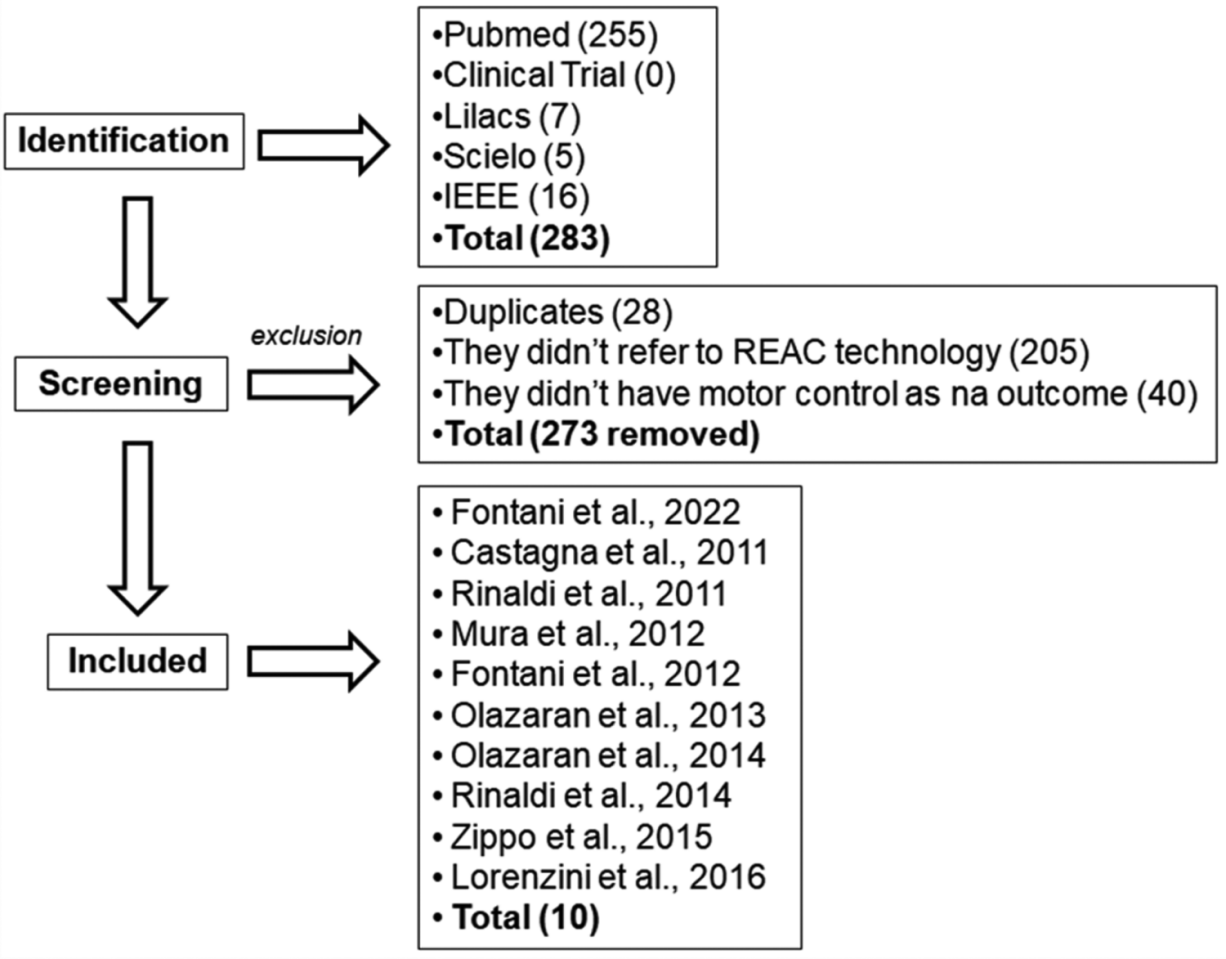
Flowchart of the identification, screening, analysis and inclusion of studies in the review process.

Through the search engine, 283 studies were found, of which 234 were excluded by duplication and title because they did not use the REAC technology. Of the remaining 49 studies, 39 were excluded because they did not present motor control as a primary or secondary outcome. As a result, ten studies were reviewed. The process of identification, screening, and inclusion of studies is summarized in Figure 1.

Out of the ten studies included in this review, eight were clinical studies and two were experimental, with animal models. Three of the clinical studies had a prospective longitudinal design, three were randomized controlled trials and one was retrospective.

The reviewed articles were published in the last 10 years, most of them between 2011 and 2015. Most of the studies were developed in Italy, specifically at the Instituto Rinaldi Fontani located in the city of Florence. Two studies were carried out in Spain at the Alzheimer's Center of the Reiná Sofía Foundation.

The characteristics of the studies regarding the type and country are given in Table 1, while their objectives and main results are in Table 2.

**Table 1:** Descriptive data of the studies included in the review.

Author/Year	Home country	Title	Study Type
Fontani et al., 2022	Italy	Long-Lasting Efficacy of Radio Electric Asymmetric Conveyer Neuromodulation Treatment on Functional Dysmetria, an Adaptive Motor Behavior	Retrospective
Castagna et al., 2011	Italy	Radio electric asymmetric brain stimulation and lingual apex repositioning in patients with atypical deglutition	Naturalistic (cross-sectional)
Rinaldi et al., 2011	Italy	Brain activity modification produced by a single radio electric asymmetric brain stimulation pulse: a new tool for neuropsychiatric treatments. Preliminary fMRI study	Prospective longitudinal
Mura et al., 2012	Italy	Preliminary pilot fMRI study of neuropostural optimization with a noninvasive asymmetric radio electric brain stimulation protocol in functional dysmetria	Prospective longitudinal
Fontani et al., 2012	Italy	Noninvasive radio electric asymmetric conveyor brain stimulation treatment improves balance in individuals over 65 suffering from neurological diseases: pilot study	Prospective longitudinal
Olazaran et al., 2013	Spain	Motor effects of REAC in advanced Alzheimer's disease: results from a pilot trial	Randomized controlled clinical trial
Olazaran et al., 2014	Spain	Motor effects of radio electric asymmetric conveyer in Alzheimer's disease: results from a cross-over trial	Randomized controlled clinical trial
Rinaldi et al., 2014	Italy	Long-lasting changes in brain activation induced by a single REAC technology pulse in Wi-Fi bands. Randomized double-blind fMRI qualitative study	Randomized controlled clinical trial
Zippo et al., 2015	Italy	Electrophysiological effects of non-invasive Radio Electric Asymmetric Conveyor (REAC) on thalamocortical neural activities and perturbed experimental conditions	Experimental *in vivo*
Lorenzini et al., 2016	Italy	REAC technology modifies pathological neuroinflammation and motor behavior in an Alzheimer's disease mouse model	Experimental *in vivo*

REAC, radio electric asymmetric conveyer.

**Table 2:** Objectives and results of the clinical and experimental studies included in the review.

Author/Year	Objectives	Results
Fontani et al., 2022	Check the duration of the NPO effect over time in functional dysmetria	REAC NPO treatment eliminated functional dysmetria in 100% of treated cases up to 18 years after protocol administration.
Castagna et al., 2011	To test the effectiveness of REAC brain stimulation in atypical swallowing.	The REAC therapy protocols applied led to normal positioning of the lingual apex and a significant improvement of the swallowing mechanism in all participants.
Rinaldi et al., 2011	Determine whether a single 500 ms REAC pulse, applied to the ear, can induce a modification of brain activity detectable by functional magnetic resonance imaging (fMRI)	A single REAC pulse reduced the amplitude of cortical activation during the proposed motor task as assessed by functional magnetic resonance imaging.
Mura et al., 2012	To evaluate the changes in functional dysmetria (FD) and brain activation observable by functional magnetic resonance imaging (fMRI) during a leg flexion-extension motor task after REAC brain stimulation.	Reduced functional dysmetria and decreased areas of motor cortical and cerebellar activation assessed by functional magnetic resonance imaging
Fontani et al., 2012	To verify the effect of a non-invasive REAC brain stimulation protocol, called neuropostural optimization (NPO), to improve balance in the elderly.	Improved postural control after REAC - NPO. Increase in general, anteroposterior and laterolateral stability.
Olazaran et al., 2013	To evaluate the effect of the NPO protocol on gait and mobility dysfunction. The secondary objectives were to evaluate the feasibility and safety of the NPO protocol and assess the potential impact on the domains of cognition and behavior.	Transient worsening of axial movements was observed immediately after NPO. There was some improvement in gait pattern and number of steps per second in the group treated with REAC – NPO.
Olazaran et al., 2014	To confirm and extend the safety and motor results of NPO in a larger sample of Alzheimer's patients, most of whom have advanced dementia, over a more extended evaluation period.	Delayed improvement in axial movements. Immediate and late improvement in the number of steps per second test after NPO.
Rinaldi et al., 2014	To evaluate in healthy adult subjects, with magnetic resonance imaging (fMRI), lasting changes in brain activation patterns after administration of a single 250-millisecond pulse delivered with REAC.	Decrease in the magnitude of cortical activation, a reduction in the conspicuity of activated áreas, and a disappearance of the thalamic activation component after a single REAC pulse - NPO
Zippo et al., 2015	Analyze spontaneous spiking activity and Local Field Potentials (LFP) before and after REAC application.	The REAC application can reorganize the level of synaptic activities and the dynamics of related neural circuits in the brain. The dynamic property of REAC maintained the electrogenic activity of the brain at appropriate intervals and induced recovery by dynamic bidirectional rearrangements.
Lorenzini et al., 2016	To explore possible mechanisms underlying the effects observed after REAC RGN-N treatment in Alzheimer's patients, the impact of specific REAC treatment in a mouse model of AD (Tg2576 mice) was investigated.	REAC RGN-N treatment modifies pathological neuroinflammation and attenuates part of the motor behavior changes observed in old Tg2576 mice. Although these results should be considered only preliminary, to the best of our knowledge, this is the first report describing a modulation of neuroinflammation in AD animal models by neurostimulator/modulatory techniques.

REAC, radio electric asymmetric conveyer; NPO, neuro postural optimization; AD, Alzheimer's disease.

The clinical studies evaluated 473 patients. The age of the research subjects was quite heterogeneous among the studies and the averages ranged from 9 to 86 years. There was also great variability in terms of clinical, functional, cognitive, and psychological characteristics. Altogether, the studies evaluated 53 healthy subjects, 91 with Alzheimer's disease, 128 patients with atypical swallowing, 12 subjects with neurological disorders (three with Parkinson's disease, three with chronic cerebral vasculopathy, four with post-stroke spastic syndrome, one with multi-infarct encephalopathy and one with ataxia caused by pontine glioma) and 189 without specification of the disease.

Details of the clinical studies with humans are presented in Table 3 and those of the experimental studies, in Table 4.

**Table 3:** Characterization of the included clinical studies regarding the sample, motor tasks, and outcomes.

Author/Year	Sample			Motor tasks	Outcomes
	Subjects	Age	Gender		
Fontani et al., 2022	Patients treated at the SONC* affiliated clinical centers	Average of 47.7 years	–	Functional dysmetria test	Functional dysmetria
Castagna et al., 2011	Patients suffering from atypical swallowing *n* = 128	9 to 27 years	60 F 68 M	Swallowing ability Positioning of the tongue	Fluorescein distribution in the mouth by the Payne Method (tongue positioning) Lower lip clamping and lowering test (swallowing mechanism)
Rinaldi et al., 2011	Healthy Individuals *n* = 10	30 to 48 years	6 F 4 M	Touching your thumb on each finger of your right hand	Cortical activation assessed by functional magnetic resonance imaging
Mura et al., 2012	Active students *n* = 10	25 to 32 years	6 F 4 M	Alternating knee flexion-extension in dorsal decubitus Functional dysmetria	Brain activation assessed by functional MRI Functional dysmetria test
Fontani et al., 2012	Patients with neurological diseases *n* = 12	Average of 74.8±5.1 years	11 F 1 M	Romberg test performed on a stabilometric computer	General, anterior, posterior, and lateral instability.
Olazaran et al., 2013	Patients with Alzheimer's disease *n* = 31	GE: 84.2±2.1 years GC: 85.4±1.2 years	24 F 7 M	Turn over in bed, rise from the supine to the sitting position and get out of the chair. Gait.	Score in the axial movement test. Score in the rating scale for gait evaluation in cognitive deterioration. Number of steps in 7 meters.
Olazaran et al., 2014	Patients with Alzheimer's disease *n* = 60	GE: 84.7±1.3 years GC: 83.5±1.6 years	52 F 8 M	Turn over in bed, rise from the supine to the sitting position and get out of the chair. Gait.	Score in the axial movement test. Score in the rating scale for gait evaluation in cognitive deterioration. Number of steps in 7 meters.
Rinaldi et al., 2014	Healthy Individuals *n* = 33	32.4 years	19 F 14 M	Simulation of walking in dorsal decubitus (flexion and extension of hip and knee with heel kept in contact with the bed)	Brain activation assessed by functional MRI Functional dysmetria test

F, female; M, male; GE, experimental group; GC, control group.

**Table 4:** Characterization of the experimental studies included in relation to the sample, motor tasks and evaluation methods.

Author/Year	Sample		Motor Tasks	Evaluation Methods
	Experimental model	Features		
Lorenzini et al., 2016	Transgenic (*n* = 12) and non-transgenic mice (*n* = 12)	Expressing the mutated human amylase precursor protein gene	Y-Maze Test Contextual fear memory conditioning	Immunoreactivity testing of representative hippocampal sections. Analysis of amyloid protein deposition by electron microscopy of slices of the cerebral cortex and hippocampus.
Zippo et al., 2015	Normal rats (*n* = 12) and experimental chronic pain rats (*n* = 15)	Chronic constriction model to generate peripheral mononeuropathy	Motor scheme test Mechanical allodynia test Heat hyperalgesia test	Postural rhythms during free walking. Patterns of paw placement during walking. Reflex responses to non-harmful stimuli.

Regarding the REAC protocols, all the clinical studies used the neuro postural optimization (NPO) protocol. Castagna et al. used the neuro psycho physical optimization (NPPO) protocol, in addition to NPO (42). The experimental studies by Zippo et al., 2015 and Lorenzini et al., 2016 used the antalgic neuromodulation and neuroregeneration protocols respectively (43) and (29). The protocols and parameters used in the reviewed studies are shown in Table 5.

**Table 5:** REAC protocols and parameters used in the studies included in this integrative review.

	Author/Year	REAC Protocol	Parameters	Application Form
Clinical studies	Fontani et al., 2022	NPO	Not Informed	Single session
	Castagna et al., 2011	NPO NPPO	NPO: single 500 ms burst at the ear scapha. NPPO: seven 500 ms bursts at seven auricular points. Frequency of 10.5 GHz and absorption rate of 7 µW/kg.	NPO: single session NPPO: 18 sessions on alternate days
	Rinaldi et al., 2011	NPO	Single 500 ms burst of 10.5 GHz at the ear scapha. Distance of 150 cm from the emitter, corresponding to an injected current density of 7 µA/cm^2^.	Single session
	Mura et al., 2012	NPO	Single 250 ms burst of 5.8 GHz at the ear scapha. Distance of 150 cm from the emitter, corresponding to an injected current density of 7 µA/cm^2^.	Single session
	Fontani et al., 2012	NPO	Single 500 ms burst at the ear pavilion. Frequency, current density, and distance to the emitter were not informed.	Single session
	Olazaran et al., 2013	NPO	Single 250 ms burst of 10.5 GHz at the ear scapha. Distance of 150 cm from the emitter, corresponding to an injected current density of 7 µA/cm^2^.	Single session
	Olazaran et al., 2014	NPO	Single 250 ms burst of 10.5 GHz at the ear scapha. Distance of 150 cm from the emitter, corresponding to an injected current density of 7 µA/cm^2^.	Single session
	Rinaldi et al., 2014	NPO	Single 250 ms burst of 10.5 GHz at the ear scapha. Distance of 150 cm from the emitter, corresponding to an injected current density of 7 µA/cm^2^.	Single session
Experimental Studies	Zippo et al., 2015	Antalgic Neuromodulation	Platinum-iridium needle placed on cervical muscles (mainly on the medial raphe of the muscle acromyotrapezius). Burst duration, frequency, current density, and distance to the emitter were not informed.	15 minutes of repeated impulses, one each 1.5 s
	Lorenzini et al., 2016	Regenerative type-N protocol (RGN-N)	2.4 GHz emission with on/off waveform modulation (750 ms on / 1500 ms off) with an electrical field of 0.1 V/m at 300 cm from the emitter.	12 hours a day for 15 days

*REAC, radio electric asymmetric conveyer; NPO, neuro postural optimization; NPPO, neuro psycho physical optimization protocol; F, frequency.

According to Rinaldi et al. the NPO protocol consists of a single radio electric burst of 500 ms at 10.5 GHz with REAC CRM devices, and of 250 ms at 5.8 GHz with REAC BENE devices (32). The radio electric burst is dispersed in the environment, while the result of its interaction with the endogenous bioelectrical activity of the subject being treated is conveyed to the area to be treated *via* the specific probe called asymmetric conveyer probe (ACP). In the REAC NPO treatment a specific ACP is positioned on a precise area of the auricular scapha. In the NPO protocol the specific absorption rate of 7 µW/kg for a current density of 7 µA/cm^2^ and a distance of 150 cm from the emitter. With these parameters, a radio electric field of approximately 20 µW/m^2^ is generated (32). The NPO protocol is normally used at the beginning of treatments with REAC in association with other protocols. According to Fontani et al., NPO improves postural attitude (38), reduces pain symptoms, and optimizes motor strategies in healthy individuals (14, 26) and also in those with motor impairment (44). The NPO thanks to the long duration of its effect (14), controllable through the stable disappearance of the functional dysmetria does not require further administrations (38).

The neuro psycho physical optimization protocol (NPPO), according to Rinaldi et al., (27), consists of seven radiofrequency pulses of 500 ms conveyed by a specific ACP at precise points of the auricle (30, 45). According to Rinaldi et al., the NPPO protocol is performed in cycles of 18 therapy sessions administered on alternate days (32). Usually, each session lasts around 5 s.

The NPPO treatment aims to reorganize the overall dysfunctional adaptive changes, also on an epigenetic basis, which are stratified during life. Furthermore, NPPO treatment is intended to prevent them. To try to achieve and maintain this goal it is necessary to repeat the treatment cycles as environmental interaction and allostatic pressure are unavoidable. The generally recommended interval between cycles of the NPPO protocol is approximately six months.

The study by Zippo et al., 2015, used the antalgic neuromodulation protocol (ANM) in animal models, with pulses repeated every 1.5 s (43). The aim of the ANM is to alleviate pain by determining and sustaining the progressive process of optimizing epigenetic adaptive responses at the level of pain circuits. Clinically, it can be applied to the specific site of pain, in the cervico-brachial region and in the cervico-dorsal-lumbar region. It is usually applied in cycles, of 18 sessions and the recommendation is to have one to three cycles/year in relation to the state of suffering and the determining cause of the pain. The tissue regeneration optimization protocol (TO-RGN) was used in the study of Lorenzini et al. and consists of a radiofrequency emission of 2.4 GHz with a very low intensity at a distance 300 cm from the emitter (29). The radio electric burst modulation is done through a waveform on/off system, where there will be 750 ms on and 1,500 ms off (25).

## Discussion

The purpose of this integrative review was to seek evidence to explain the mechanisms of action of REAC technology to modulate neurophysiological aspects involved in motor control. In addition, it was also aimed to identify in the studies the protocols, parameters, and forms of application of the REAC used to act in the mechanisms of motor control. For this, we sought clinical and experimental research in the databases that used REAC and investigated motor outcomes. As a result, it was possible to select 10 studies, 8 clinical and 2 experimental.

### The role of endogenous bioelectricity in neuronal physiology

According to Frohlich (46) synchronized neuronal activity in the cerebral cortex generates weak electrical fields that are routinely measured in humans and animal models by electroencephalography and local field potential recordings (46). This neuronal synchronization was analyzed in the study by Zippo et al., 2015, which used local field potential records to determine the coherence of neuronal activity before and after the use of REAC. The authors verified that after REAC treatments there seems to be a reduction in local field potentials which can be interpreted as a temporal redistribution of synaptic signals related to the peaks of neuronal firing rate observed in the studied animals (43).

Until recently, these endogenous electric fields were considered an epiphenomenon of brain activity, but it is currently understood that this energy is able to change protein metabolism, the process of cell differentiation, electrical and chemical synaptic signaling, the process of inflammation, tissue repair and pain perception (47). Some studies have shown that possibly endogenous electric fields may play an active role in neuronal circuits since neuron signaling depends on the generation and transmission of transient electrical impulses that represent the fundamental unit of information in the brain (48, 49).

So, is the presence of endogenous electric fields just an epiphenomenon? Or can it be considered a necessary event for the amplification of neuronal communication?.

According to Fries et al. (50), the neural information processing model is based on the notion that changes in the electrical potential inside the neurons in relation to the constant electrical potential outside the neuron determine the membrane voltage and, therefore, the functional state of individual neurons. Consequently, this compartmentalization of electric fields has been postulated as a form of cellular communication and transmission of neuronal signals (51).

Therefore, endogenous bioelectric fields seem to be fundamental to aiding synchronization in neuron communication, especially when it comes to motor control. Motor control, therefore, depends on the coordinated and stable cortical activity that is partly due to the electric field generated by the simultaneous activity of the cells that make up the brain tissue, both neurons and cells of the glial system (12).

Therefore, the normality of the bioelectric field in the central nervous system depends on the morphological and functional integrity of the cells and, thus, neurological and psychiatric diseases are related to changes in bioelectricity, which contribute to stereotyped responses of brain functions (46, 51, 52). This leads to clinical, psychological, and functional manifestations that characterize a given disease (17, 18, 20, 28, 37).

### The effect of REAC technology on endogenous bioelectricity

Three studies included in this review (12–14) showed that the NPO protocol can modify brain activation in healthy individuals. Mura et al. (2012) showed that REAC could reduce activation in cortical motor areas using magnetic resonance imaging (fMRI) during the sit and stand task. Furthermore, the study's results showed the disappearance of right thalamic activation and a decrease in the activation of the cerebellar vermis and the pontine and midbrain regions (13).

Thus, reduced brain activation in the motor cortex detected by fMRI may be related to a more efficient motor strategy with fewer brain areas being activated, supposedly only those which are strictly necessary. In fMRI, brain (cortical) activation during motor activity is represented by increased or decreased BOLD signals (53). This signal appears in the resonance image due to the magnetic properties of hemoglobin that determine distortions in the magnetic field, especially when the hemoglobin is deoxygenated (54).

Therefore, reductions in the BOLD signal can represent a decrease in brain activity determined by the reduction of local blood flow or an increase in the neuronal metabolic rate that determines the presence of deoxygenated hemoglobin (53, 55). Whatever the reason for the reduction of the BOLD signal, the findings suggest that the REAC technology seems to induce activation of regions that are strictly necessary for the accomplishment of the motor task, excluding or inhibiting secondary neuronal circuits that would not be essential for the motor execution (12–14).

It is important to highlight that reducing brain activation means, in this case, a “functional optimization” of the neuronal circuits as, to perform the same task, the subject uses fewer neural resources, increasing the system's efficiency.

One of the studies included in this review (43) sought to analyze the firing rate and Local Field Potentials (PFL) before and after the application of the REAC protocol in normal mice and those with chronic neuropathy. According to the authors, the results obtained were interpreted as a “functional optimization” of the brain structures that govern the coordination of motor control and balance (43). This is because it was possible to observe that REAC reduced the firing rate of the thalamic and cortical regions both in spontaneous activities and in the tactile evoked stimulation of the animals. It was also observed that the firing rate of the thalamic and cortical circuits was significantly reduced in the animals with chronic pain (43).

According to Berge, the neuronal processing of pain hinders the segregation and integration of information in the thalamic cortical circuits due to the sensory overload that determines changes in the electrical fields around the neurons responsible for this processing (56). Pain processing, therefore, determines increases in the firing rates of higher centers and changes in the synchronization of local field potentials. In the study by Zippo et al., present in this review, it was possible to verify that REAC was able to increase the synchronization of potentials of local fields, possibly indicating that the technology can rebalance the endogenous bioelectric fields (43). The segregation and integration of information are related to neuronal communication, and REAC seems to increase the efficiency of this system, especially in animals with chronic pain (57).

The studies of Rinaldi et al. (2011) and (2014) found similar results in brain activation, especially the disappearance of the activation signal of the thalamic component after the NPO protocol of REAC (12, 14). It is known that the thalamus is considered a fundamental processing center of the central nervous system, responsible for the integration and reorganization of stimuli from the periphery, brainstem, and higher centers to filter information and modulate signals for a motor or autonomic response to be reproduced more efficiently (58). The reduction in thalamic activity possibly indicates an adjustment of the somatosensory processing involved in the planning and executing a motor task. It is as if the thalamus better integrates sensory information and filters stimuli from the periphery or brainstem better before sending the information to the motor cortex (59).

The reorganization of endogenous electric fields seems to be responsible for these effects on the nervous system, as it is known that these fields are fundamental for synaptic communication, especially when it comes to motor control (46). Lorenzini et al. demonstrated that REAC treatment improved locomotion in aged mice carrying the human amyloid precursor protein mutation (model for Alzheimer's disease) (29). In Alzheimer's disease, the deposition of amyloid protein in the hippocampus, thalamus, and motor cortex hinders the communication of neurons responsible for integrating decisive information for the processing of locomotion. It probably alters the local electric field (60). According to Lorenzini et al., REAC treatment increased glial fibrillary acidic protein, a molecule part of the cytoskeleton of several cells, including astrocytes. In this study, REAC favored the increase in the proliferation of astrocytes around the amyloid plaques determined by the higher expression of the glial fibrillary acidic protein. In vitro and *in vivo* studies (49) indicate that astrocytes may play a role in removing debris from injury and amyloid plaque deposits (61).

Thus, it can be assumed that the application of REAC technology protocols can optimize neuronal communication by favoring the containment or removal of amyloid plaques. Thus, the establishment of a balanced electric field possibly influences the activity of neurons inducing the activation of cells that would be responsible for transmitting information (12, 13). This mechanism may explain the results of the study by Olazarán et al., 2013 and 2014, which showed that REAC delayed axial movements in patients with Alzheimer's disease. In addition, in this study, there was an immediate and delayed improvement in the steps per second and gait test after using the NPO-REAC (3, 31).

These explanations may be related, for example, to the results of one of the studies included in this review: Castagna et al. aimed to test the effectiveness of REAC brain stimulation in atypical swallowing (42). Atypical swallowing is mainly characterized by the malfunction of the tongue and other muscles during the swallowing reflex, often caused by neurological or structural dysfunctions of the stomatognathic system (62). The motor response of the masticatory muscles in this situation consists of the interposition of the tongue between the teeth or lips since the possible hypotonia of the masticatory muscles makes the mouth open at the time of the oral phase of swallowing (63).

According to Hebling et al., stress and anxiety seem to be related to swallowing abnormalities, especially in the oropharyngeal phase of the process. The oropharyngeal phase consists of the sequence of events that begins with the voluntary contraction of the muscles of mastication and gradually passes into involuntary control. This process is mediated by mechanisms that involve integrating information from the swallowing center in the brainstem with the motor cortex through the basal ganglia (64). Therefore, changes in endogenous electrical fields determined by anxiety disorder (18), for example, can influence the synchronization of information necessary for normal swallowing control. Barcessat et al. demonstrated that REAC technology appears to be effective in reducing anxiety and stress. Thus, if we consider anxiety and stress as factors involved in atypical swallowing, we can assume that the bioelectrical modulation provided by REAC may favor the correction of the swallowing mechanism in patients with atypical swallowing (5).

According to the developers of the REAC treatment, Rinaldi and Fontani, environmental stress is responsible for causing morpho-functional changes in the central nervous system capable of inducing the development of functional dysmetria (38). This motor behavior is defined as a deviation from perfect bilateral symmetry. It can be considered a manifestation of the deterioration of the allostatic mechanisms of development influenced by genetic alterations and environmental stress factors (65). The study by Fontani et al., identified that all patients who received REAC NPO treatment showed the disappearance of functional dysmetria up to 18 years after administration of the protocol (37, 38). This phenomenon can be explained by the functional optimization of neuronal communication, where an increase in the efficiency of the activation pattern substantiates the participation of neural circuits strictly necessary to perform the motor task (12–14). In this context, functional dysmetria has been an evaluation parameter inherent to the treatment process with REAC since the evidence of its disappearance possibly indicates the effectiveness of the treatment.

## Conclusion

Clinical studies that evaluated the effect of REAC on motor tasks showed that the technology is able to improve motor response through functional optimization of neural circuits. The experimental studies included in this review indicated that REAC technology influences glial cell activity and improves neuronal synchronization by reducing local field strengths. These findings may be a promising basis for elucidating the mechanisms involved in the modulation of technology-induced neuronal bioelectrical activity.
